# What We Learned From Big Data for Autophagy Research

**DOI:** 10.3389/fcell.2018.00092

**Published:** 2018-08-17

**Authors:** Anne-Claire Jacomin, Lejla Gul, Padhmanand Sudhakar, Tamas Korcsmaros, Ioannis P. Nezis

**Affiliations:** ^1^School of Life Sciences, University of Warwick, Coventry, United Kingdom; ^2^Earlham Institute, Norwich Research Park, Norwich, United Kingdom; ^3^Gut Microbes and Health Programme, Quadram Institute, Norwich Research Park, Norwich, United Kingdom

**Keywords:** autophagy, big data, proteomics, bioinformatics, transcriptomics

## Abstract

Autophagy is the process by which cytoplasmic components are engulfed in double-membraned vesicles before being delivered to the lysosome to be degraded. Defective autophagy has been linked to a vast array of human pathologies. The molecular mechanism of the autophagic machinery is well-described and has been extensively investigated. However, understanding the global organization of the autophagy system and its integration with other cellular processes remains a challenge. To this end, various bioinformatics and network biology approaches have been developed by researchers in the last few years. Recently, large-scale multi-omics approaches (like genomics, transcriptomics, proteomics, lipidomics, and metabolomics) have been developed and carried out specifically focusing on autophagy, and generating multi-scale data on the related components. In this review, we outline recent applications of *in silico* investigations and big data analyses of the autophagy process in various biological systems.

## Introduction

To maintain their homeostasis, cells require an appropriate balance between anabolism and catabolism. There are two main degradative processes for intracellular components in eukaryotic cells: autophagy and the ubiquitin-proteasome system (Dikic, 2017). Whereas the ubiquitin-proteasome system is primarily known for its implication in the turnover of short-lived proteins, autophagy contributes to the degradation of long-lived cytosolic proteins, as well as large protein complexes and organelles (Yin et al., 2016). Autophagy plays a fundamental role in maintaining a healthy cell, and defective autophagy process has been associated with a broad range of pathologies. As such, it is not surprising that more and more studies are focusing on understanding the molecular mechanisms of autophagy. With the advent of the post-genomics era, a growing number of studies make use of the analysis of massive molecular data sets for a more comprehensive understanding of autophagy processes related to basal condition and disease/infection pathologies.

In the present review, we go through examples on the latest research in understanding autophagy mechanisms based on the analysis of a large volume of data from omics studies, and how these findings have been gathered in databases freely available to the scientific community.

## Autophagy: from physiology to pathology

### Physiological roles of autophagy

Autophagy is a survival mechanism greatly conserved among every eukaryotic organism. Autophagy functions essentially as an adaptive response to stress, particularly in the condition of nutrient deprivation, allowing for cell and organism survival. When nutrient resources are restricted, cells are able to break down and reprocess all sort of macromolecules including proteins, lipids, and carbohydrates which can then be reused as essential building blocks for the synthesis of new macromolecules and the production of energy (Kaur and Debnath, 2015).

Although most of the knowledge on the autophagy process was generated from studies performed in different conditions of stress, it is now broadly acknowledged that constitutive degradation of cytoplasmic contents by basal autophagy under favorable growth conditions also plays an essential role in cell physiology. A basal level of autophagy is essential for the maintenance of cellular homeostasis in post-mitotic cells (for instance, neurons or hepatocytes) that cannot dilute their deleterious components through division. As such, autophagy facilitates the disposal of supernumerary or damaged proteins and organelles before they become toxic to the cell (Pankiv et al., 2007; Kirkin et al., 2009; Okamoto et al., 2009; Richter et al., 2016). A broad range of studies has revealed that basal autophagy decline is often associated with pathologies such as neurodegeneration, cancer and inflammation.

Because of the proficiency of autophagy to target large organelles such as mitochondria for degradation, it is not surprising it is largely exploited by the innate immune system to fight microbial invasion (Gomes and Dikic, 2014; Randow and Youle, 2014). The term xenophagy is used to refer to the autophagic degradation of bacteria, viruses, and parasites which are strict intracellular pathogens. Besides, autophagy has other roles in immunity such as the control of pro-inflammatory responses and antigen presentation by macrophages (Crotzer and Blum, 2009).

### The plurality of autophagic processes

**Figure 1:**
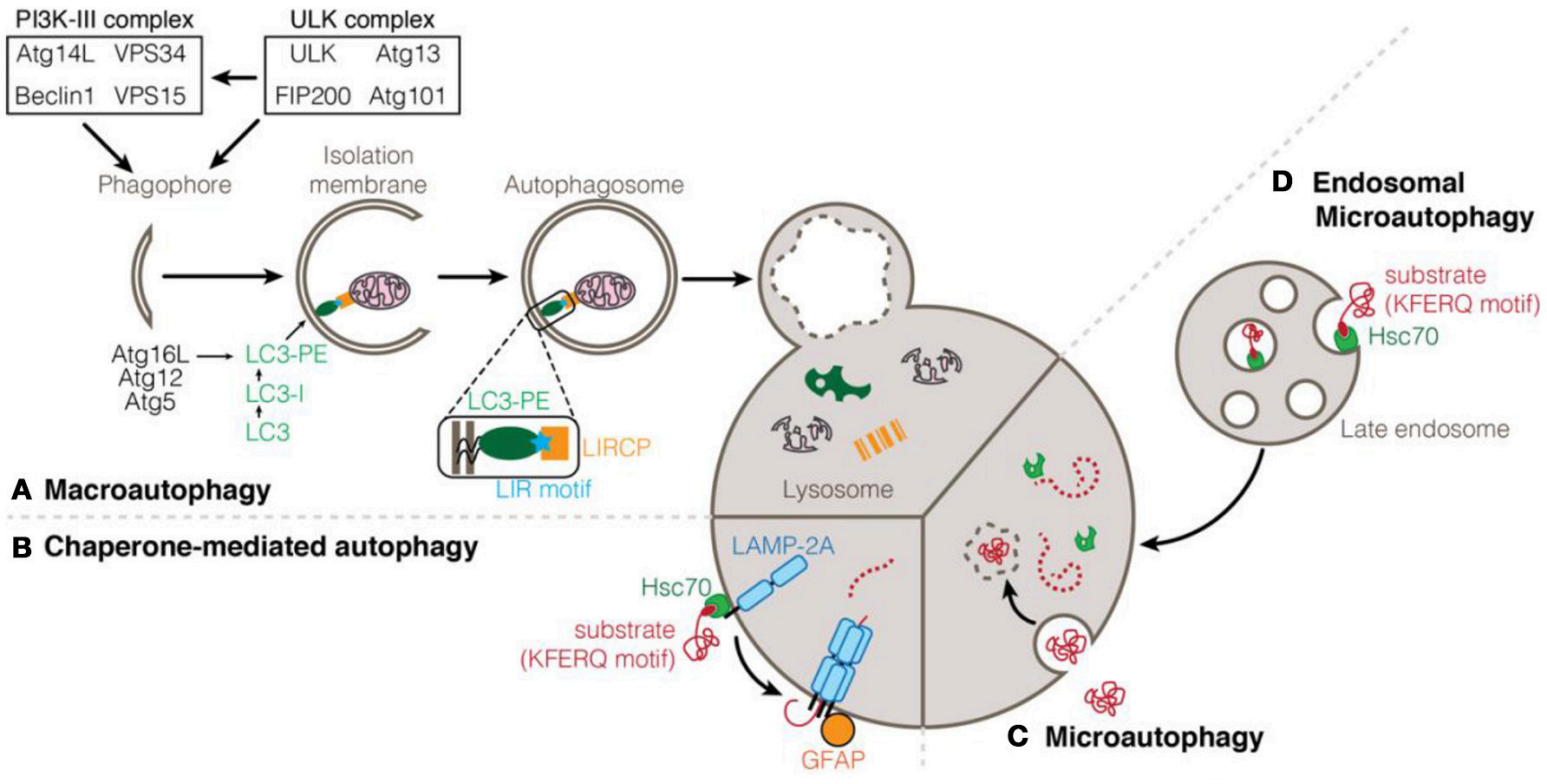
Types of autophagy processes. **(A)** Macroautophagy is induced by the activation of the ULK1 and PI3K-III complexes. Modification of LC3/ATG8 proteins with phosphatidylethanolamine (PE) and anchorage to the membrane of the elongating autophagosome depends on the Atg12-Atg5-Atg16L conjugation system. LC3 can interact with cargoes and selective autophagy receptors via a LIR motif. Enclosed autophagosome eventually fuses with the lysosome for the degradation and recycling of its content. **(B)** Chaperone-mediated autophagy (CMA) consists in the translocation of proteins into the lysosome through pores formed of LAMP-2A protein stabilized by phosphorylated GFAP. **(C)** Microautophagy consists in the internalization on cytoplasmic components into the lysosome by direct invagination of the lysosomal membrane. **(D)** Endosomal-microautophagy depends on the isolation of cytosolic protein in the late endosome before being addressed to the lysosome for degradation. Both CMA and endosomal-microautophagy rely on the chaperone protein Hsc70 that can bind to substrate proteins containing a KFERQ motif.

Autophagy relates to a set of catabolic processes for the delivery of cytosolic components to the lysosome for degradation. To date, three types of autophagy processes have been described, depending on the manner by which the cargo reaches and is delivered to the lysosome: macroautophagy, (endosomal-) microautophagy, and chaperone-mediated autophagy (Figure 1). Initially, autophagy was described to be non-specific and degrading material in bulk with minimal regulation. However, recent evidence described autophagy as a tightly regulated process which makes use of a multitude of accessory proteins in order to identify and transport the cargo to the lysosome (Yu et al., 2018).

In chaperone-mediated autophagy (CMA), cytosolic proteins interact with the chaperone heat shock 70 kDa protein 8 (HSPA8/HSC70) via their pentapeptide KFERQ; the chaperone in turn binds to the lysosomal-associated membrane protein 2A (LAMP2A) (Agarraberes and Dice, 2001). The recognition and binding of the substrate to monomers of LAMP2A induces its multimeric assembly, which is essential for the translocation of unfolded substrate proteins into the lysosome (Gough and Fambrough, 1997; Salvador et al., 2000; Bandyopadhyay et al., 2008). Although the regulation of CMA has not been fully identified yet, it appears that the phosphorylation status of the regulatory protein GFAP contributes to the stability of the CMA translocation complex (Bandyopadhyay et al., 2010; Figure 1).

Microautophagy is the newest of the autophagy process sub-type that has been identified and to this date remains poorly understood. Early studies conducted in yeast have suggested that, during microautophagy, cytosolic content is sequestered by invagination of the lysosomal membrane that pinches off into the lumen (Müller et al., 2000). The inability to detect direct invagination of the lysosome has considerably delayed the investigation of microautophagy in higher eukaryotic organisms. It was only recently that studies have demonstrated the existence of a process similar to yeast microautophagy which occurs at the level of late endosomes instead of lysosomes. As such, this process is being referred to as endosomal-microautophagy (eMi) (Sahu et al., 2011; Mukherjee et al., 2016). While eMi seems to contribute mainly to in-bulk degradation of cytosolic substrates, it appears that proteins can be selectively targeted in a similar way than CMA with the implication of the chaperone HSC70 and the presence of KFERQ motifs on substrate proteins (Tekirdag and Cuervo, 2017; Figure 1).

Last but not least, the best-characterized form of autophagy is macroautophagy (mostly simply referred to as autophagy; Figure 1). It is characterized by the engulfment of intracellular material in a double-membrane vesicle called the autophagosome. Mature autophagosomes are transported along microtubules and ultimately fused with lysosomes leading to the degradation of the autophagosome contents (Yin et al., 2016). Initiation of autophagosome formation depends on the activation of the ULK and PI3K-III complexes. The PI3K-III complex is itself activated by the ULK complex and is responsible for the generation of PI3P (phosphatidylinositol-3-phosphate), essential for autophagy induction (Feng et al., 2014). Although initially thought to be nonselective, the delivery of cargo to the autophagosome occurs in a specific and controlled manner. At the membrane of the autophagosome, ATG8-family proteins serve as anchor points for the recruitment of the cargo and autophagy machinery. ATG8 proteins are bound to the autophagosomal membrane after their conjugation to phosphatidylethanolamine (PE) via two ubiquitin-like conjugation systems that involve several ATG proteins (Nakatogawa et al., 2009). Specialized selective autophagy receptors are required for the proper targeting of the cargo. Many of those autophagy receptors (also known as LIR-containing proteins, LIRCPs) share common features such as a Ubiquitin Binding Domain (UBD) which allows them to bind to polyubiquitinated cargo, or a LC3-interacting region (LIR) motif which allows for the binding of the receptor to ATG8 proteins at the autophagosome membrane (Pankiv et al., 2007; Behrends and Fulda, 2012; Birgisdottir Å et al., 2013; Jacomin et al., 2016; Gatica et al., 2018).

### Pathologies associated with autophagy dysfunctions

Extensive research over the past years has unraveled a central role of autophagy not only for cellular homeostasis but also for various pathological conditions. Autophagy dysfunction has been observed during aging, and several genetic alterations in cancer, neurodegenerative and immune-related diseases have been associated to autophagy and autophagy genes (Zhou and Zhang, 2012; Carroll et al., 2013; White, 2015). While it is widely accepted that autophagy is involved in disease development and progression, its exact roles often appear to be controversial across similar studies, highlighting that its implication is most likely to be context-dependent. For instance, the cytoprotective function of autophagy is believed to have tumor-suppressive potential at the early stages of tumorigenesis, and that loss of autophagy can be associated with increased risk of cancer (Roy and Debnath, 2010). Nonetheless, autophagy has also been shown to allow premalignant cells to escape genotoxic stress and inflammation, thus promoting tumorigenesis (Hu et al., 2012; Kubisch et al., 2013).

During aging, however, activation of autophagy is widely accepted as being beneficial to counteract mechanisms involved in the development of neurodegenerative diseases (Nakamura and Yoshimori, 2018). Current studies are exploring how autophagy induction could constitute a strategy for the prevention and treatment of neurodegenerative diseases. Indeed, in healthy cells, autophagy allows for the elimination of ubiquitinated protein aggregates and non-functional organelles. Accumulation of protein aggregates inside neuronal cells is a hallmark of neurodegenerative and age-associated diseases (including, but not restricted to, Alzheimer's and Parkinson's diseases, ALS, Huntington's disease; Del Roso, 2003; Bartlett et al., 2014; Tóth et al., 2014; Menzies et al., 2017). Moreover, numerous proteins implicated in autophagy or lysosomal function were found to be mutated in neurodegenerative diseases (Menzies et al., 2017).

## Web-based resources related to autophagy

**Figure 2:**
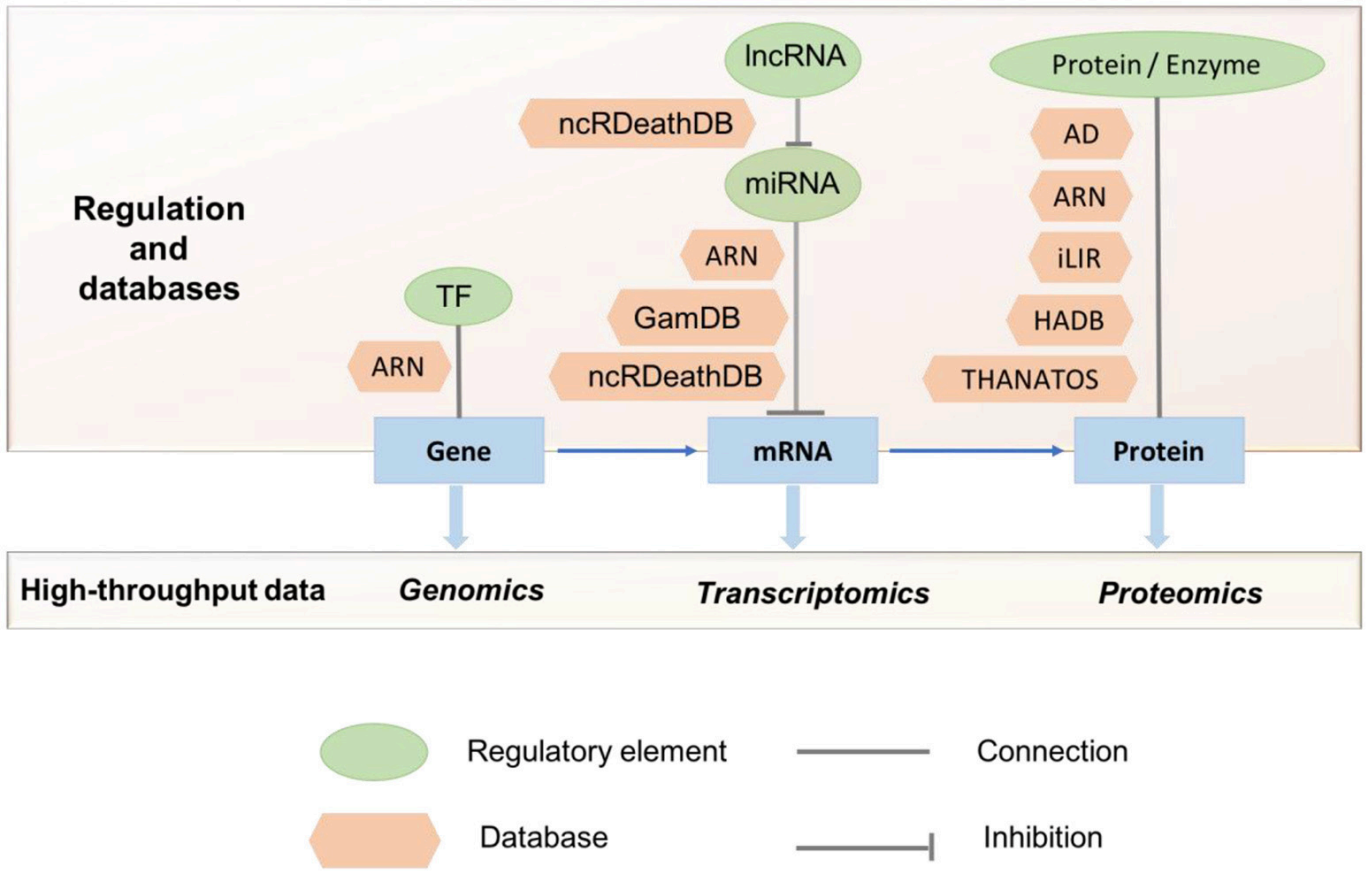
Studies using multi-omics data to understand autophagy and its regulation. Currently available autophagy-related databases highlighting the different stages of regulation and high-throughput data are shown. TF, Transcription Factor; ARN, Autophagy Regulation Network; GamDB, Gerontology-Autophagic-MicroRNA Database; AD, Autophagy Database; HADB, Human Autophagy Database; THANATOS, THe Autophagy; Necrosis, ApopTosis OrchestratorS.

With the increasing interest in the field of autophagy, the past 15 years have seen a rise of publicly available autophagy-related resources. These resources provide access to a broad range of data types and offer functionalities for the identification and characterization of proteins involved in various autophagic processes. Currently, there are several databases containing information on the autophagy components and the molecular players which modulate the process from different regulatory layers (transcriptional, post-transcriptional, translational etc.; Table 1, Figure 2). The following section aims to highlight the different servers that allow for either the identification of autophagy-related proteins and genes or the characterization of features that could link proteins to autophagy.

**Table 1:** Currently available autophagy-related resources.

**Database**	**URL**	**Description**	**References**
Autophagy Regulation Network	http://autophagyregulation.org/	Autophagy core proteins and their regulators with experimentally verified and predicted interactions in human.	Türei et al., 2015
The Human Autophagy Database	http://autophagy.lu/	Manually curation of autophagy proteins and their regulators.	
The Autophagy Database	http://www.tanpaku.org/autophagy/	Provides information about proteins related to autophagy, including protein structure data in 41 species.	Homma et al., 2011
The Autophagy Ontology	http://atgo.ucsd.edu/	Ontology of functions related to autophagy, explains and presents the hierarchy of functions in autophagy in yeast and human.	Kramer et al., 2017
ELM	http://elm.eu.org	Annotation and detection of eukaryotic linear motifs (ELMs).	Gouw et al., 2017
Gerontology-Autophagic-MicroRNA Database	http://gamdb.liu-lab.com/index.php	Experimentally validated interactions between miRNA and genes or proteins in gerontology-related disorders manually curated from the literature focusing on autophagy.	Zhang et al., 2016
hfAIM	http://bioinformatics.psb.ugent.be/hfAIM/	Identification of LIR/AIM motifs in protein sequences	Xie et al., 2016
iLIR	http://repeat.biol.ucy.ac.cy/iLIR	Identification of LIR/AIM motifs in protein sequences and providing a PSSM score indicating the significance of the hits.	Kalvari et al., 2014
iLIR database	http://ilir.uk/model	A database listing all the putative LIR-containing proteins from 8 model organisms; GO analysis.	Jacomin et al., 2016
iLIR@viral	http://ilir.uk/virus/	Database of all the putative LIR-containing proteins in viruses.	Jacomin et al., 2017
ncRDeathDB	http://www.rna-society.org/ncrdeathdb	Collection of programmed cell death associated non-coding RNAs (including microRNA, long noncoding RNA/lncRNA and small nucleolar RNA/snoRNA).	Wu et al., 2015
SLiMSearch	http://slim.ucd.ie/ slimsearch/	Search function in given protein(s) for a specific consensus motif for the discovery of novel motif instances.	Krystkowiak and Davey, 2017
THANATOS	http://thanatos.biocuckoo.org/	Manually curated collection of experimentally verified regulators during programmed cell death in eukaryotes.	Deng et al., 2018

### Prediction of Atg8-family interacting proteins

Atg8 family proteins are a central component of the autophagic machinery. Their covalent anchorage to lipid membranes is crucial for the expansion and closure of the autophagosome. They are also essential for the selective degradation of cargoes. In the past decade, Atg8-family protein interactomes have been extensively studied, and the interaction of a number of proteins with the Atg8 homologs is mediated by a pentapeptide known as LIR/AIM/LRS (LC3-interacting region, Atg8-interacting motif, LC3 recognition sequence; Pankiv et al., 2007; Ichimura et al., 2008; Noda et al., 2010). The presence of such short linear motifs has provided a reliable way to predict the interaction between any given protein of interest and the Atg8-family members. As such, tools have been developed for the identification and prediction of LIR motifs.

#### The iLIR server

The iLIR server scans an input protein sequence for the presence of putative LIR motifs (Kalvari et al., 2014). The results are sorted either as an extended LIR-motif (xLIR), or “canonical” LIR motif (WxxL), where “x” can be any amino acid with the only restrictions for W (W/F/Y) and L (L/I/V) positions. Besides, each motif is associated with a position-specific scoring matrix (PSSM) based on experimentally validated LIR motifs; the higher the PSSM score, the higher the confidence in the predicted motif to be involved in the interaction with Atg8-family proteins. Finally, iLIR also overlays the LIR motif results with intrinsically disordered protein regions as predicted by the ANCHOR package. Such protein segments are likely to form stabilizing interactions upon binding. The combination of a high PSSM scoring (>13) xLIR motif that overlaps with an ANCHOR region should provide reliable predictions. The main limitation of this resource is that it cannot predict any non-canonical LIR motifs. The iLIR server is freely available online at the URL *http://repeat.biol.ucy.ac.cy/iLIR* and *http://ilir.warwick.ac.uk/search.php*.

#### The high-fidelity AIM system (hfAIM)

The high-fidelity AIM system (hfAIM) is a server used for the prediction of putative LIR/AIM motifs in sequences of interest using five regular expression motifs (Xie et al., 2016). As a proof of concept, the authors have utilized hfAIM to identify potential LIRs in PEX proteins from several model organisms. Using a cell biology approach, they have identified PEX10 as containing at least one functional LIR motif and to interact with Atg8 in the plant model *Arabidopsis thaliana*. The hfAIM resource is available online at the URL: *http://bioinformatics.psb.ugent.be/hfAIM/*

#### The eukaryotic linear motif resource (ELM)

The Eukaryotic Linear Motif (ELM) resource is a database, and web server focused on short linear motifs (SLiMs) (Gouw et al., 2017). SLiMs are short protein sequences that can be involved in protein-protein interactions and the modifications of protein sequences. SLiMs are implicated in almost all cellular processes, including cell signaling, trafficking, protein stability, cell-cycle progression. ELM was first released in 2003 and has been regularly updated since then. This resource has incorporated 7 entries related to LIR motifs: 4 ELM classes (added in May 2014) and 3 ELM candidates still being evaluated. ELM lists 24 instances of LIR motifs, including one instance identified in the protozoan parasite *Plasmodium falciparum*, one instance for the nematode *Caenorhabditis elegans* widely used as a model organism, 22 instances are related to human proteins (21 are canonical LIR motifs, 1 correspond to the non-canonical LIR motif from NDP52 necessary for its interaction with LC3C). ELM is maintained by several European groups coordinated by the Gibson group at the European Molecular Biology Laboratory (EMBL). ELM is publicly available online at the URL *http://elm.eu.org*.

#### SLiMSearch

SLiMSearch is a short, linear motif (SLiM) discovery tool which allows the user to use a motif consensus to search a proteome and discover putative novel motif instances (Krystkowiak and Davey, 2017). Consensus matches are annotated with experimental, proteomic and genomic data; annotations include the description of domains and structures, post-translational modification, single-nucleotide polymorphism, and isoforms. SLiMSearch also provides functional enrichment and evolutionary analysis tools. It is possible to analyse GO terms, keywords and enrichment of interacting partners. SLiMSearch supports a range of species, including bacteria, plants and fungi, and is freely available online at http://slim.ucd.ie/slimsearch/.

### Autophagy networks and databases

#### The autophagy regulatory network (ARN)

ARN is a multi-layered molecular interaction database related to autophagy in human, providing users with validated and predicted interaction between proteins, transcription factor and gene, and miRNA and mRNA (Türei et al., 2015). All the interactions in the database have been gathered and extensively manually curated from 26 resources. The interaction network was built around a core of 38 autophagy proteins and gathers over 397,000 interactions. All autophagy components and regulators have been linked to major signaling pathways.

The download functionality gives the user the flexibility to use locally the entire ARN data or a part of it in a broad range of formats (CSV, Cytoscape).

The Autophagy Regulatory Network resource is publicly available online at the URL http://autophagyregulation.org.

#### iLIR database and iLIR@viral

The iLIR prediction server has been used to develop two databases: the iLIR database and iLIR@viral. The iLIR database provides a list of all the putative canonical (xLIR and WxxL motifs) LIR-containing proteins identified using the iLIR resource (see before) in the proteomes of 8 model organisms, combined with a Gene Ontology (GO) term analysis (Jacomin et al., 2016). The iLIR@viral database focuses on the identification of putative LIR-containing proteins in viruses known to be linked to autophagy (Jacomin et al., 2017). The databases are accessible online at the URLs http://ilir.uk/model/ and http://ilir.uk/virus/, respectively.

#### The thanatos database

THANATOS (THe Apoptosis, Necrosis, AuTophagy OrchestratorS) is a database that integrates sequence data curated from the literature and related to programmed cell death in eukaryotes (Deng et al., 2018). The database was built based on the manual curation of the literature to identify autophagy-related proteins in the most commonly used models, followed by ortholog searches in 164 eukaryotes. As of the last update in May 2017, the THANATOS database contains information about 191,543 proteins from 164 eukaryotes, which are potentially associated with autophagy and cell death pathways. The web interface allows the user to search the database and retrieve data using keywords and browsing by species and cell death type. Information related to posttranslational modifications on query sequences is also available. The THANATOS database is publicly available online at the URL http://thanatos.biocuckoo.org/.

#### The human autophagy database (HADb)

The Human Autophagy Database lists over 200 human genes and proteins involved directly or indirectly with autophagy that have been manually collected from the literature. For each entry, HADb provides information on the sequence, transcripts and isoforms. Links to external resources and relevant literature are also available for each entry. HADb is publicly available online at the URL http://autophagy.lu/.

#### The autophagy database

The Autophagy Database is a freely accessible web resource aiming at providing up-to-date information about proteins related to autophagy, including protein structure data (Homma et al., 2011). This resource was last updated in January 2017 and contains information regarding 582 reviewed protein entries. The database also provides additional data regarding orthologous/homologous proteins of the reviewed entries. In addition to offering the possibility to look through the available data, the server also provides the possibility for the user to search the database using keywords and BLAST homology based on query sequences against the database entries. The Autophagy Database is publicly available online at the URL http://www.tanpaku.org/autophagy/.

#### The ncRNA-associated cell death database (ncRDeathDB2.0)

The noncoding RNA (ncRNA)-associated cell death database (ncRDeathDB) documents over 4,600 ncRNA-mediated programmed cell death entries (Wu et al., 2015). The ncRDeathDB gathers published data that describe the roles of ncRNAs (including microRNA, long noncoding RNA/lncRNA and small nucleolar RNA/snoRNA) in programmed cell death. The current version of ncRDeathDB summarizes data from 12 species with 4,615 ncRNA-mediated programmed cell death entries: 2,403 entries associated with apoptosis, 2,205 entries associated with autophagy and 7 entries associated with necrosis. The ncRNA-associated cell death interactions resource is publicly available online at the URL http://www.rna-society.org/ncrdeathdb.

#### AutomiRDB

AutomiRDB is a web resource that combines information related to experimentally identified human miRNAs and their autophagic target genes/proteins in different types of cancers (Chen et al., 2015). An extensive text-mining of the literature was conducted to identify all the known autophagy-related miRNAs. A combination of several miRNA predictive databases was used to predict candidate miRNAs targeting the 49 cancer-related autophagy genes/proteins identified by the authors. The database gives access to 493 miRNAs related to autophagy, 90 targeted autophagic genes or proteins, and 18 types of cancers. Hyperlinks of targets and diseases are provided for easy access to other databases, such as UniProt, OMIM. AtomiRDB is available at the URL http://www.chen-lab.com/index.php

#### GAMDB

GAMDB (Gerontology-Autophagic-MicroRNA Database) is an open-access knowledge depository which contains 836 microRNAs associated with autophagy, 197 targeted genes or proteins, and 56 diseases related to aging (Zhang et al., 2016). The database was developed based on published articles and public online databases. Experimentally validated autophagy-related miRNA and targeted autophagic genes/proteins in gerontology-related diseases were manually curated from the literature. In the GAMDB website, the user can use microRNAs as keywords to conduct a query and retrieve detailed information. The users also can upload the novel microRNA information through a submission interface. The GAMDB is available at the URL http://gamdb.liu-lab.com/index.php

## Omics studies in the autophagy field

Read-outs from high-throughput, omics approaches provide us with context-specific and dynamic information on the state and regulation of cellular processes, such as autophagy. Given that “omics” based investigations are a pivotal area of current research to provide a more holistic understanding of biological systems, such approaches have guided our insights into the regulation of autophagy. In this section, we seek to provide a few examples of studies to illustrate how omics approaches can be used for gaining a better understanding of autophagy processes.

### Genomics and transcriptomics

Genomic approaches including gene mapping and DNA sequencing study the structure and function of the genome, while transcriptomic approaches provide information about the transcriptional changes in the organism on the RNA level. Yang et al. examined autophagy components in 84 species (eukaryotes, eubacteria, and archaebacteria) to discover similarities and differences in terms of the various autophagy phases and the regulatory components involved. Proteome-level data from UniProt was used to identify homologous proteins involved in autophagy across species. Finally, proteins which exist in different taxa were identified with the “hmmsearch” tool in HMMER3 (*http://hmmer.org/*). Phylogenetic trees using 16S/18S rRNA were reconstructed as a reference to compare the similarity among autophagy genes in different species. Normalized symmetric tree similarity algorithm was used to measure the similarity of the constructed phylogenetic trees. The resulting analysis has revealed that the core autophagy proteins are present in most of the investigated species with the difference being a fewer number of core proteins in plants and protists. The main difference lies in the vesicle elongation, and maturation phase of the autophagy pathway wherein most plants are characterized by a lack of some of the ATG5–ATG12 conjugation-related proteins. Some of the prokaryotic homologs of the core autophagy proteins were also suggested to play different roles in the process upon comparison with the eukaryotic species. For instance, ATG11 has an essential role in selective autophagy regarding the yeast organism, in contrast to Archaebacteria this protein is liable for DNA repair and reorganization of chromosomes. By collating the phylogenetic trees, the most significant similarity was found to be among proteins which are responsible for the autophagosome nucleation and ATG9 cycling. In terms of selective autophagy, the phylogenetic trees were quite different from each other. The analysis also highlighted the fact that parasitic organisms such as *Entamoeba histolytica* and *Plasmodium yoelii* which have relatively fewer ATG proteins still exhibited autophagic activity (Yang et al., 2017).

Foldvari-Nagy et al. published a similar observation when they used both BLAST and HMMER methods to identify autophagy-like proteins in 40 non-unikont parasitic protists (e.g., *Trypanosoma* and *Plasmodium*). According to a comprehensive computational analysis, *Atg1* and genes encoding its induction complex were found to be lacking in the genomes of all the studied species. As an alternative induction of autophagy, < 20 species contained Atg6/Beclin1, in the other remaining investigated non-unikont parasites, this protein does not appear in the genome. In this case, there is no evidence which could provide a clue on how autophagy is induced in these species (Foldvari-Nagy et al., 2014).

The relationship between autophagy and the cellular homeostasis in the nervous system is poorly discovered, based on our knowledge malfunction of autophagy causes protein aggregation and neurodegeneration. Lipinski and colleagues investigated the transcriptional level alterations between healthy aging and Alzheimer disease (AD), and they found up-regulated autophagy in brain samples from AD patients (compared to normal brain samples). Based on these observations, it was suggested that the up-regulated autophagy signatures in the AD patients could be a compensatory mechanism in order to remove the accumulated protein aggregates (Lipinski et al., 2010).

Besides its importance in neuronal functions, autophagy also influences the identity and function of myeloid cells as well. Huang et al. examined how the expression pattern of autophagy genes is changing when myeloid cells differentiate to monocytic and granulocytic cells. RNA-Seq data from CD34+ hematopoietic stem and pluripotent cells (HSPCs) exposed to monocytic and granulocytic induction helped discover the relationships between autophagy and the differentiation of HSPCs. Differentially expressed genes involved in the autophagy process were inferred by combining the observed transcriptional changes and annotations/regulator information obtained from the Autophagy Database, Autophagy Regulatory Network (ARN) and Human Autophagy Database (HADb). Based on the analysis of the temporal gene expression data using a standard clustering algorithm, 22 autophagy genes were found to be significantly altered during the monocytic and granulocytic differentiation process of myeloid progenitors into monocytes and granulocytes. The results suggested that autophagy is essential to maintain the balance between different states (quiescence, self-renewal, and differentiation) in myeloid cells (Huang et al., 2018).

Autophagy has also been investigated using multi-omics approaches in the context of its role in host-pathogen interactions. Lu et al. studied how autophagy influences the homeostasis in lungs and resistance to influenza infection by comparing the transcriptional profiles of lung macrophages derived from normal mice and mice deficient in multiple autophagy components including Atg5, Atg7, Atg14, Epg5, and FIP200. RNA-seq analysis revealed the dysregulated pathways in autophagy-deficient macrophage cells. It turned out that *Epg5* is essential for basal autophagy and autophagosome formation and is supported by increased inflammation and lethal influenza virus infection resistance in the lung upon its absence (Lu et al., 2016).

### Proteomics

Mass spectrometry (MS)-based proteomics is an invaluable way for studying protein-protein interactions, protein expression, subcellular localization, and post-translational modifications. Given the increased interest in the identification of new players in the regulation of autophagy, it is not surprising that MS-based proteomics approach has been widely used and has successfully contributed to advancing the knowledge on autophagy.

One of the most straightforward applications of MS-based proteomics is the comparison of whole cell proteomes between autophagy-deficient cells and autophagy-competent cells. As such, a study conducted by Zhuo and colleagues in atg7^−/−^ MEFs has identified 66 upregulated and 48 downregulated proteins (Zhuo et al., 2013). This study led to the identification of F-actin and showed that it plays a role in both basal and starvation-induced autophagy. Analysis of the whole neosynthesized proteome after induction of autophagy was performed by adding an azide methionine mimetic, azidohomoalanine (AHA), into the culture media. AHA is subsequently incorporated into newly synthesized proteins, which can be further enriched by affinity isolation before analysis by LC-MS/MS. This innovative approach has been successfully used in HeLa cells, allowing for the profiling of 711 newly synthesized proteins. Several hits were further validated and characterized; for instance, ATP5B, RACK1, and SLC25A3 proteins were identified as playing a role in the promotion of autophagy (Wang et al., 2016).

The formation of the autophagosome is one of the hallmarks of the autophagy process. Due to its importance, studies focused specifically on the proteome of the autophagosome have been performed. The characterization of proteins associated with the autophagosomes were carried on isolated autophagosomes. Dengjel and colleagues used a density gradient to separate fractions containing the autophagosomes from MCF7 breast cancer cells. Selected fractions were analyzed by LC-MS/MS, and for nonspecific co-purifying proteins, the PCP-SILAC method was applied (Dengjel et al., 2012). A total of 728 putative autophagosome-associated proteins were identified from the analysis of autophagosomes isolated from cells subjected to amino acid starvation or treatment with either the autophagy-inducer rapamycin that inhibits the mTOR complex 1, or the lysosomal inhibitor concanamycin A. Only 94 proteins were common to all stimuli and some of them were previously identified by independent studies that aimed to identify autophagosome-membrane associated proteins (Gao et al., 2010; Øverbye et al., 2014). The poor overlap between these three studies may be due to the difference in stimuli used, cell types, and purification and MS analysis differences.

Another advantage of the MS-based proteomics resides in the high-throughput identification of protein-protein interaction partners. In 2010, an extensive study of the autophagy network by Behrends and colleagues provided a global view of the autophagy interaction landscape in basal condition. In this study, 32 human proteins related to autophagy or vesicle trafficking were used as prey in the 293T cell line, and the immune complexes were analyzed by MS. A total of 751 interactions among 409 candidate interacting proteins were revealed. The study focused on the protein partners of the six ATG8-family proteins. Thirty-eight ATG8-interacting proteins were tested *in vitro* for their binding to ATG8 proteins. Up to 60% of the interactions were reduced or lost when the LIR-docking site on ATG8 proteins was mutated, indicating that a substantial proportion of ATG8-interacting proteins do so through an LDS/LIR-dependent binding (Behrends et al., 2010). More recently, Le Guerroué and colleagues used a state-of-the-art proximity-proteomics-based autophagosome content profiling to identify the interacting partners of the ATG8 proteins. Screening for the interactors of all six ATG8 proteins in mammals, they identified 1,147 proteins with considerable overlap across GABARAP and LC3 family members and among GABARAP and LC3 subfamilies. This approach led them to identify the mitochondrial protein MTX1 that is targeted by LC3C and p62 to maintain basal mitochondrial homeostasis through a piecemeal mitophagy pathway (Le Guerroué et al., 2017).

### Metabolomics and lipidomics

Metabolomics is a recently emerging field aimed at the systemic profiling of the metabolites, which are the small molecule intermediates and products of metabolism. Metabolomics is a powerful approach to complement other “omics” as metabolite profiles and concentration reflect the functional and physiological state of cells/organisms. Studies of the metabolome are based on two key techniques: nuclear magnetic resonance spectroscopy (NMR) and mass spectrometry (MS) (Markley et al., 2017). Because autophagy is tightly associated with the cell stress status, it is not surprising that autophagy-related metabolomes will be subject to changes depending on the nature of the stresses happening in the cells (Stryeck et al., 2017).

Autophagy is strongly activated by starvation conditions characterized by low levels of glucose or amino acids. When glucose levels are high, ATP is converted into cAMP and is itself further degraded into AMP. As such, a high AMP:ATP ratio reflect a high glucose level; while a reduced AMP:ATP ratio is typical of starvation conditions when glucose levels are low. Thus, glucose, ATP, cAMP, and AMP are appropriate read-outs used in autophagy-related metabolomics studies (Stryeck et al., 2017).

Mutations in the *RAS* oncogene control tumor growth and RAS-associated tumors heavily rely on autophagy. Because cancer usually depends on high glucose level, Lashinger and colleagues have used a mouse model system to investigate the effect of caloric restriction and autophagy on the development of RAS-driven tumors (Lashinger et al., 2016). They have shown that combining autophagy blockade (Atg5-deficient mice) to caloric restriction was sufficient to reduce the tumor volume significantly. Using NMR, they observed that caloric restriction induced a switch away from glucose metabolism, characterized by a reduction of glucose, amino acids, and tricarboxylic acid cycle intermediates, and upregulation in ketone bodies. Similar observations made by Gaglio et al. wherein blocking autophagy using the inhibitor chloroquine caused massive cell death of NIH-RAS cancer cells *in vitro*. However, using chloroquine *in vivo* did not produce any notable effect on highly aggressive NIH-Ras xenografts. Nevertheless, changes in the metabolome of the tumors were observed after treatment, suggesting that RAS-driven tumors have the ability to adapt to environmental modifications and metabolic stress using metabolic rewiring and alternative pathways (Gaglio et al., 2016).

Another *in vitro* study, conducted by Redmann et al. on cultured primary rat cortical neurons from E18 embryos used HPLC-MS metabolomics approach to investigate the impact of lysosome inhibitors on bioenergetics and metabolism (Redmann et al., 2017). Notably, they showed that autophagy inhibition decreased metabolites of the TCA cycle, essentially downstream the citrate synthase and those linked to glutaminolysis. Their results implicate that inhibitors of autophagy impact on cellular bioenergetics and metabolism in primary neurons, probably due to decreased mitochondrial quality control.

Lipidomics is a sub-category of metabolomics that focuses on the identification and quantification of cellular lipids. While it has been described that changes in the cellular level of ceramides—a family of lipids—can affect autophagy, little is known about the regulation of these lipids by autophagy itself. A recently published study by Alexaki and colleagues sought to evaluate the implication of autophagy in the regulation of ceramides in the liver, as autophagy is essential in this organ to maintain homeostasis and prevent metabolic diseases. To this end, the authors used HPLC-MS to identify and quantify the ceramide species in the liver from wild-type and *Atg7*^−^^/−^ mice. They observed that ceramides are significantly increased in autophagy-deficient livers, which is correlated with an increase in serine palmitoyltransferase, the enzyme that catalyzes the *de novo* synthesis of sphingolipids, included ceramides. Based on their observations, the authors suggest that autophagy may contribute to the regulation of serine palmitoyltransferase level by targeting the degradation of the protein subunits or endoplasmic reticulum membranes containing excessive ceramide near serine palmitoyltransferase (Alexaki et al., 2014). In an *in vitro* study, Tharkeshwar and colleagues have focused their interest on the lipid composition of isolated lysosomes in the context of Niemann-Pick disease type C1 (NPC1) deficiency. Niemann-Pick disease type C (NPC) is a severe inherited lysosomal storage disorder, most often originating from the loss-of-function of NPC1. Their lipidomics analysis on lysosomes isolated using superparamagnetic iron oxide nanoparticles (SPIONs) revealed the build-up of several species of glycerophospholipids and other storage lipids in lysosomes of NPC1-deficient cells (Tharkeshwar et al., 2017).

## Integration of omics dataset to understand autophagy better

Integration of omics datasets with signaling and regulatory networks is used to study biological processes and their regulation on a systems-level. Recent studies supported by advances in experimental omics technologies and computational data integration approaches have shed light on the mechanistic and regulatory aspects of autophagy. Here, we list a few examples from various areas using different omics approaches.

Despite the dramatic increase in the volume of generated information on autophagy such as the identification of core components, their regulators etc., the complexity of the autophagy process itself as well as the functional hierarchy in the biological system makes data representation and analysis a challenging task. Some of these issues were overcome by using Gene Ontology (GO) which assigns genes and their encoded products with specialized ontology terms capturing the entire range of the functional hierarchy. Kramer et al. recently developed a general framework—AtgO (http://atgo.ucsd.edu/index.html), to explain and present the hierarchy of autophagy functions in yeast. Published omics data was combined with a newly generated genetic interaction map targeted at autophagy. It contains almost 500 genes which could be related to the process of autophagy according to the literature and results from experiments. Using protein sequences and structure details, researchers revealed interactions between proteins or genes, co-expression levels and similarities between genes. The AtgO process schema was applied to human data as well which resulted in the Human Autophagy Ontology database (hAtgO) containing 1,452 genes and 1,664 terms describing autophagy based on the expression profile of genes, interactions between proteins, co-localization, etc. (Kramer et al., 2017).

Due to the homeostatic role of autophagy and its dysregulated/deregulated status in many chronic diseases, exploring the connections between cancer and autophagy is a growing research area. While on one hand autophagy suppresses tumorigenesis, cancer cells also activate the process to avoid the stress and up-regulate growth and tumor aggression (Lorente et al., 2018). Autophagy strongly influents cancer so that modulation of this process has been identified as a potential target for cancer therapy (Kubisch et al., 2013). Omics data integration is widely used to investigate the genomic events and their interactions, as well as the potential regulatory mechanisms affected in cancer (Sompairac et al., 2018). In their recent study, Chen et al. focussed on identifying genes related to breast cancer and its multiple subtypes at the genomic level with an integrated bioinformatics approach. They used the Least Absolute Shrinkage and Selection Operator (LASSO) method which is designed for -omics data integration. By bringing together three different data types namely mRNA expression (DNA microarray), DNA methylation (Illumina Methylation Assay), and copy number alteration (GenomeWideSNP_6 array) data provided by The Cancer Genome Atlas database (TGCA—*https://cancergenome.nih.gov/*), they identified the regulators of BECN1, a core autophagy component, which has an inhibitory effect on tumor formation (Chen et al., 2017).

Aneuploidy, an unbalanced karyotype when the cell contains extra chromosomes, is an often discovered disorder during cancer. Stingele et al. used data from comparative genomics, transcriptomics, and proteomics to investigate the molecular manifestations of aneuploidy. In more details, they discovered those proteins and transcripts which are encoded on the extra chromosomes. Focussing on the fate of transcripts and proteins encoded on the extra chromosomes, the gene copy number, mRNA and protein levels were measured with array comparative genomic hybridization, microarray analysis, and mass spectrometry, respectively. The integrative analysis revealed that mRNA levels increased with gene copy numbers, but the relative abundance of proteins was significantly reduced. Interestingly, autophagy and lysosome-mediated degradation were found to be consistently up-regulated suggesting a higher demand for autophagy in aneuploid cells possibly due to an elevated requirement to degrade the extra-coded proteins (Stingele et al., 2013).

*Arabidopsis thaliana* is a versatile plant model organism in which autophagy plays a key role in various processes from immune responses to environmental adaptation. Autophagy is also activated during leaf senescence and exposure to external stressors such as pathogen attack, starvation etc. Masclaux-Daubresse et al. investigated the effect of autophagy alterations by studying the transcriptomic and metabolomic signatures of autophagy mutants. Gas chromatography-mass spectrometry (GC-MS) was used to measure small sized molecules and liquid chromatography-mass spectrometry (LS-MS) and enzymatic assays for complex molecules. Microarray analysis followed by quantitative reverse transcription polymerase chain reaction (qRT-PCR) measured the transcriptomic level changes. Integration of metabolomics and transcriptomics data from the rosette leaves elucidated the pleiotropic effect of autophagy activity on cellular homeostasis and revealed the dependence on autophagy of various metabolic pathways. Based on the results from the transcriptomics datasets, many genes related to plant stress response were also overrepresented upon autophagy malfunction in the mutants (Masclaux-Daubresse et al., 2014).

## Promising omics approaches—the future of systems-level autophagy studies

### Single-cell analysis in autophagy research

Single cell sequencing gives us an inside view at a cellular level by enabling the measurement of genomic and transcriptomic readouts from individual cells. This possibility has opened up investigations into biological processes and functions in different cell types—which has hitherto not been possible at high-throughput. Several studies which look into the role of autophagy in numerous cell-types have also recently been published.

Due to its central and homeostatic role in cellular physiology and metabolism, autophagy interacts with a vast number of other processes in the cell. Filippi-Chiela et al. probed the interplay between autophagy and senescence using human glioma cells which were modified to represent the DNA damage-induced senescence using a relevant model. Using single-cell sequencing, they demonstrated the differences in the correlation between the two processes in glioma cells compared to the cell-type non-specific population. While at the population cell level, autophagy and senescence were negatively correlated, data from the human glioma cells indicated otherwise with a complete absence of such a correlation between the two processes (Filippi-Chiela et al., 2015).

Xu et al. revealed a similar study to investigate the differential dynamics between autophagy and apoptosis using single-cell sequencing in tandem with experimental live microscopy imaging of cells from multiple cell lines. The cells were exposed to various autophagy eliciting stimuli including starvation and mTOR inactivation. Autophagy was induced to different degrees suggesting autophagy to be a pathway with a wide magnitudinal response window. However, under all the tested stimuli, apoptosis also was induced in a binary fashion—either completely induced or absent, thus representing a bimodal response mechanism. Using fluorescent reporters and live cell imaging, the dynamic responses of individual cells were tracked to reveal that autophagy preceded apoptosis. However, in those cells which mounted a very strong autophagic response, there was a time-lag before the activation of the cell death. This was further verified by the upregulation of apoptosis in a cell line deficient in *atg5* which is essential for autophagic activity (Xu et al., 2013).

Hematopoietic stem cells (HSCs) are a lineage of stem cells which are present in both early embryonic and adult hematopoietic organs with the capability to renew themselves and differentiate into multiple lines of blood cells. Due to the essential role of autophagy in self-renewal and differentiation in embryonic hematopoiesis, Hu et al. investigated its role in five populations of mice cells related to HSC formation during the process of mouse embryogenesis by measuring gene expression using single-cell RNA sequencing. The results from the study revealed an increase in the transcriptional level of various autophagy component encoding genes in endothelial cells which were classified as pre-HSCs. This observation was synchronous with the down-regulated Notch signaling thus suggesting that autophagic activity could play a significant role in the formation of HSCs during the gestation period (Zhou et al., 2016; Hu et al., 2017).

Although the single-cell analysis is a rapidly evolving approach, there are two main challenges which could be solved in the future, namely the integration and interpretation of omics data. In general, the single-cell analysis covers various steps (isolation, sorting, library preparation, and sequencing) when the cell is separated from its bulk environment. On one hand, while the examination of the cell in an isolated milieu can lead to data loss, on the other hand, the cell is a dynamic structure where the molecular states will differ temporally across different sampling points. A future goal will be to improve the *in situ* analysis techniques in fixed cells or tissues and develop the analysis of live imaging. The second challenge is to carry out multi-omics profiling which provides insights into cell regulatory mechanisms, such as autophagy. Examining the biological processes by using the techniques mentioned above facilitates to understand the complexity of human health/disease in order to develop more effective therapies (Yuan et al., 2017).

### Patient-derived samples

Compared to various *in vitro* models, investigations on patient-derived samples are desirable since it provides researchers with the necessary basis to understanding disease mechanisms for translational purposes as well as personalizing treatments. Martínez-Pizarro et al. investigated the role of endoplasmic reticulum (ER) stress and autophagy in fibroblasts derived samples diagnosed with homocystinuria—a disease characterized by defective cysteine metabolism and manifested by neurological and behavioral anomalies. Earlier studies reported upregulated levels of reactive oxygen species and apoptotic activity in fibroblasts derived from homocystinuria patient biopsies. However, the exact mechanisms in terms of the molecular pathologies underlying the disease were not explained. In the study by Martínez-Pizarro et al. the authors used a combination of techniques such as PCR, Western blotting, microscopy and cytosolic Ca^2+^ imaging followed by *in vitro* assays to first identify and then verify the mechanistic roles of the identified molecular mediators. Gene and protein expression profiling revealed the induction of a number of proteins related to ER stress and calcium signaling. In addition, autophagy was also found to be activated along with mitophagy-mediated degradation of modified mitochondria. Treatment with antioxidative agents inhibited autophagy suggesting that reactive oxygen species affect autophagy which otherwise would impart a protective role under homeostatic conditions (Martínez-Pizarro et al., 2016).

Inflammatory bowel diseases (IBD) represent a group of intestinal disorders which are characterized by chronic inflammation. The most common forms of IBD are Crohn's disease (CD) and ulcerative colitis (UC): while CD affects any part of the gastrointestinal tract, UC results in inflammation in the colon and the rectum. Silverberg and colleagues collected samples from healthy and diseased patients suffering from CD or UC. They extracted RNA from peripheral blood mononuclear cells and examined the post-transcriptional regulation of autophagy. The study revealed that there are differences in miRNA expression between the healthy and diseased samples. miRNAs are small non-coding RNA molecules which bind to mRNAs thereby silencing their transcription. miRWalk 2.0 software was used to predict miRNA-mRNA interactions and thereafter with GO and pathway analysis, the dysregulated pathways were identified and analyzed. As a result, the authors observed that the most of the differentially expressed miRNAs can effect on the autophagy pathway confirm the significant effect of miRNA-mediated modulation on autophagy (Mohammadi et al., 2018).

### High throughput screening (HTS) and high content analysis (HCA)

With interest in identifying new regulators of autophagy in different cell types or different stress conditions, High Throughput Screening (HTS) and extraction of vast amount of data from automated image analysis have gained in popularity over the past decade. Indeed, High Content Analysis (HCA) allows for the unbiased quantitation of phenotypic images through the development of automated image acquisition and analysis. Most autophagy-focused HCA screenings rely on the quantitation of autophagosomes using fluorescent-tagged LC3. In order to identify new autophagy-related genes, He and colleagues used a library of cDNA to investigate the implication of 1,050 genes of unknown function in autophagy. The overexpression of the transmembrane protein TM9SF1 resulted in the accumulation of autophagosomes and general increase of the autophagy flux in HeLa cells. Silencing of TM9SF1 using si-RNA impaired starvation-induced autophagy. In addition, TM9SF1 localized to the autophagosome and lysosome, suggesting a direct implication of this protein in the autophagy process, although its molecular mechanism and function remain unclear (He et al., 2009). Focusing on bacterial effector associated with Crohn's disease a screen was performed by overexpression 224 GFP-fused proteins from Adherent Invasive *E. coli* (AIEC) strain LF82 in HeLa cells and monitoring the induction of autophagy using mCherry-LC3 (Collins and Huett, 2018). The analysis also concentrated on overall cellular and nuclear morphology and actin cytoskeleton. This work did not provide any molecular mechanism, but instead was made available to the scientific community as all the raw images and original analysis files can be downloaded for further analysis (Collins and Huett, 2018). Another recent study focused on the identification of modulators that are specific for p62-mediated selective autophagy (Hale et al., 2016). Instead of using tagged-LC3 in their primary screening, Hale and colleagues used GFP-p62/SQSTM1 and lysosomal marker LAMP2 to screen si-RNA targeting over 12,000 genes in U2OS fibroblast cell line. The mean intensity in GFP-p, as well as GFP-p62 colocalization with LAMP2 were used to assess the induction of autophagy. From the 12,000 genes initially screened, 10 hits were eventually selected and validated to induce an upregulation of the autophagy flux when knocked down (Hale et al., 2016). Altogether, these three studies showed that image-based high content analysis is a robust strategy for the identification of new modulators of autophagy, that can be applied to a broad range of conditions.

Giving the fact that autophagy is related to a broad range of pathologies, targeting autophagy machinery components and regulators could be an appealing alternative to classical chemotherapeutic agents. To this end, in order to fill the gap in the number of autophagy inhibitors and potential therapeutic agents, Peppard and collaborators designed a phenotypic, cell image-based assay for small molecules that affects the accumulation of autophagosomes in starved cells expressing GFP-LC3 (Peppard et al., 2014). Over 240,000 compounds were screened, leading to the identification and qualification of about 400 active molecules.

However, stimulating autophagy may constitute a promising approach to prevent or treat some pathologies in aging-related diseases. Chiang and colleagues sought to identify new autophagy inducers that disrupt the binding between Beclin-1 and Bcl-2 (Chiang et al., 2018). For their primary screening of about 300,000 small molecules, they developed an assay using a split-luciferase to measure the interaction between these two key regulators in cells. Selected compounds were then tested *in vitro* using a Beclin-1/Bcl-2 AlphaLISA assay to measure the interaction between the two proteins. This two-step screen led to the identification of three active molecules that induce autophagy.

## Outlook

Autophagy is one of life's fundamental processes. Recent research has indicated roles for autophagy in an increasing number of pathologies, from bacterial and viral infections to cancer, and more recently in neurodegenerative and other age-related diseases. The importance and significance of the autophagy process was highlight very recently with the Nobel Prize award to Prof. Yoshinori Ohsumi for his pioneering studies revealing the mechanisms of autophagy in baker's yeast 30 years ago (Tsukada and Ohsumi, 1993). Big data and omics approaches provide us with the key to further elucidate the complex autophagy network and its integration with other cellular networks in the context of both health and disease.

## Author contributions

A-CJ and LG wrote the manuscript with input from PS. TK and IN edited the manuscript.

### Conflict of interest statement

The authors declare that the research was conducted in the absence of any commercial or financial relationships that could be construed as a potential conflict of interest.
